# Urinary cell cycle arrest biomarkers TIMP-2 and IGFBP7 for the assessment of acute kidney injury in dogs with pyometra

**DOI:** 10.3389/fvets.2026.1788906

**Published:** 2026-03-10

**Authors:** Larissa A. do N. Braz, Suellen R. Maia, Beatriz Gasser, Nathan da R. N. Cruz, Alef Winter Oliveira Alvarenga, Lara Vilela Soares, Ricardo A. R. Uscategui, Andrigo B. de Nardi, Leandro Z. Crivellenti

**Affiliations:** 1 Department of Clinical and Veterinary Surgery, School of Agricultural and Veterinary Sciences, São Paulo State University (Unesp), Jaboticabal, Brazil; 2 Department of Veterinary Clinics, School of Veterinary Medicine and Animal Science, São Paulo State University (Unesp), Botucatu, Brazil; 3 Instituto de Ciências Agrárias, Universidade Federal dos Vales do Jequitinhonha e Mucuri (UFVJM), Unaí, Brazil; 4 Mestrado e Doutorado Profissional em Produção e Sanidade Animal (PGPSA) – Instituto Federal Catarinense, Santa Rosa do Sul, SC, Brazil; 5 Graduate Program in Animal Science, Veterinary Teaching Hospital, University of Franca (Unifran), Franca, Brazil; 6 Graduate Program in Veterinary Science (PPGCV), College of Veterinary Medicine and Animal Science (FMVZ), Universidade Federal de Uberlândia (UFU), Uberlândia, Brazil; 7 Departamento de Sanidad Animal, Facultad de Medicina Veterinaria y Zootecnia, Universidad del Tolima, Ibagué, Colombia

**Keywords:** dogs, pyometra, renal biopsy, urinary biomarkers, veterinary nephrology

## Abstract

Acute kidney injury (AKI) is associated with increased morbidity and mortality and may develop secondary to systemic inflammatory conditions. Conventional biomarkers, such as serum creatinine and urinary gamma-glutamyl transferase (GGT), often fail to detect early renal injury. Cell cycle arrest biomarkers, including tissue inhibitor of metalloproteinase-2 (TIMP-2) and insulin-like growth factor-binding protein 7 (IGFBP7), have shown potential for early AKI detection in critically ill human patients; however, evidence in veterinary medicine remains limited. This prospective cross-sectional blinded study evaluated whether urinary TIMP-2 and IGFBP7 reflect histopathological renal injury severity in 27 clinically non-azotemic female dogs with pyometra and systemic inflammatory response syndrome (SIRS). Renal tissue samples were collected during ovariohysterectomy and graded according to tubular, glomerular and interstitial lesions. Animals were categorized as having discrete, moderate or severe injury based on cumulative lesion scores. Urinary TIMP-2 and IGFBP7, either alone or normalized to urinary creatinine, were compared with serum creatinine, symmetric dimethylarginine (SDMA) and protein-to-creatinine ratio (UPC). All dogs were non-azotemic at presentation. Tubular injury was the most prevalent histopathological finding. Conventional biomarkers did not differentiate lesion severity, whereas urinary TIMP-2 and IGFBP7 showed a significant association with increasing renal injury grade. Principal component analysis supported these findings. These results suggest that urinary TIMP-2 and IGFBP7 can detect renal lesions before azotemia and may serve as promising biomarkers for the identification of pre-azotemic renal injury in dogs with pyometra.

## 1 Introduction

Acute kidney injury (AKI) is a clinically relevant syndrome resulting from renal insult, leading to a rapid decline in glomerular filtration rate and often accompanied by electrolyte imbalance, nitrogenous waste accumulation, endocrine dysfunction and acid–base disturbances (1, 2). AKI may occur even in the absence of azotemia, particularly during early stages, which complicates diagnosis and delays intervention (3, 4). Conventional indicators such as serum creatinine and urine output are poorly sensitive for early detection and may remain unchanged until more than 75% of renal function is compromised (2, 5). In veterinary medicine, this limitation is especially relevant, as structural renal injury may already be present during subclinical phases, before conventional biomarkers become abnormal. Therefore, the identification of reliable biomarkers capable of detecting renal injury during its or pre-azotemic stages remains an important objective in both clinical and research settings.

Pyometra is a prevalent uterine inflammatory condition in dogs and is associated with systemic inflammatory response, hemodynamic disturbances and oxidative stress, which may contribute to AKI even in clinically non-azotemic animals (6–8). Although not primarily classified as a renal disease, pyometra has been shown to induce structural kidney alterations, making it a clinically relevant model to investigate or pre-azotemic renal injury under conditions of naturally occurring systemic inflammation. Additionally, the collection of renal tissue during indicated ovariohysterectomy provides an ethically acceptable opportunity to evaluate histopathological renal lesions without the need for additional invasive procedures performed exclusively for research purposes (6, 9–11).

A range of biomarkers, including N-Acetyl-*β*-glucosaminidase (NAG), kidney injury molecule-1 (KIM-1), interleukins 6 and 18 (IL-6, IL-18), neutrophil gelatinase-associated lipocalin (NGAL), cystatin C (CysC), proenkephalin (PenK) and urinary dickkopf-3 to urinary creatinine ratio (uDKK3:uCr), have been evaluated for the detection of early AKI (12–14). However, their applicability in veterinary practice remains limited due to restricted availability or inconsistent correlation with biopsy-confirmed renal lesions, which represent the current gold standard for AKI diagnosis (10, 15, 16). Moreover, most of these biomarkers primarily reflect functional or inflammatory alterations and do not consistently demonstrate a direct association with histopathological lesion severity, particularly in clinically non-azotemic animals.

Recently, urinary biomarkers associated with cell cycle arrest and indicating renal cell stress, tissue inhibitor of metalloproteinase-2 [TIMP-2(u)], and insulin-like growth factor-binding protein 7 [IGFBP7(u)], have been applied in human patients in intensive care units to predict AKI development (17–19). These two proteins are involved in cell cycle arrest during the G1 phase, which occurs if cellular DNA damage is detected (20, 21). This is a self-protection mechanism to prevent damaged cells from continuing to divide, which could lead to further damage (20). Both are expressed throughout the body, especially in renal tubular cells. During early tubular stress or injury, increased urinary concentrations of TIMP-2(u) and IGFBP7(u) can be detected, potentially reflecting subclinical structural damage before measurable changes in conventional renal biomarkers occur (21).

In human medicine, the Nephrocheck® immunoassay test (Astute Medical, San Diego, CA, United States) allows rapid and accurate assessment of urinary TIMP-2 and IGFBP7 concentrations to predict the early development of AKI (17, 18). This test generates a quantitative risk index [(TIMP-2)·(IGFBP7)], which has proven to be a reliable predictor of AKI in critically ill patients (17). Although these cell cycle arrest biomarkers have shown promising and advantageous results compared to creatinine in human medicine, veterinary research remains limited to a few studies that reported increased urinary concentrations in dogs with AKI (22, 23). Importantly, these studies did not include histopathological confirmation of renal injury, and it therefore remains unclear whether elevated urinary concentrations reflect true structural renal damage or merely transient functional alterations. To date, no veterinary study has directly correlated urinary TIMP-2 and IGFBP7 concentrations with histopathological renal lesion severity in clinically non-azotemic dogs with systemic inflammation.

We hypothesized that urinary TIMP-2 and IGFBP7 concentrations correlate with the histopathological severity of renal injury in clinically non-azotemic dogs experiencing systemic inflammation, potentially reflecting or pre-azotemic structural damage. Therefore, this study aimed to assess the association between these urinary biomarkers and histopathological renal lesion severity at the time of pyometra diagnosis, and to compare their diagnostic performance with conventional renal indicators, including serum creatinine [Cr(s)], symmetric dimethylarginine [SDMA(s)], and the urinary protein-to-creatinine ratio (UPC), using histopathology as the reference standard.

## 2 Materials and methods

### 2.1 Ethics committee

The research protocol was approved by the Ethics Committee on Animal Utilization (CEUA) under protocol numbers 1855/21 and 4979280318. All procedures were conducted following national animal welfare regulations, and informed consent was obtained from the animal owners prior to inclusion.

### 2.2 Patients

A total of 27 client-owned bitches diagnosed with pyometra were prospectively enrolled. Only non-azotemic animals (serum creatinine ≤ 1.6 mg/dL at admission) were included. Diagnosis was confirmed through clinical examination, ultrasonographic assessment and microbiological analysis. Inclusion criteria consisted of a compatible clinical history (such as recent estrus or previous progestogen administration), presence of vaginal discharge and abdominal distension. Dogs with concurrent systemic diseases, including neoplastic, chronic or endocrine conditions, were excluded to minimize confounding factors.

All dogs included met the criteria for Systemic Inflammatory Response Syndrome (SIRS) (34). SIRS diagnosis was based on at least three of the following: heart rate >120 bpm, respiratory rate >20 breaths/min, body temperature >39.2 °C or <38.1 °C, white blood cell count >16,000/μL (leukocytosis) or <6,000/μL (leukopenia), ≥3% band neutrophils, and a documented infectious focus.

This was a prospective cross-sectional study using a convenience sample; no formal sample size calculation was performed.

### 2.3 Laboratory analysis

Urinalysis was performed on samples obtained via ultrasound-guided cystocentesis prior to any treatment. Specific gravity was measured using a refractometer (ATAGO, Tokyo, Japan), and chemical analysis was performed using commercial reagent strips (LABTEST Uriquest® Plus). Microscopic sediment evaluation followed centrifugation (800 G for 5 min). Urinary protein and creatinine concentrations were quantified using the pyrogallol red method (Sensiprot kit, LABTEST) and an alkaline picrate assay, respectively, with subsequent calculation of the urine protein-to-creatinine (UPC) ratio.

### 2.4 Renal biomarkers

Global blood cell counts, including red and white blood cells and platelets, were performed using an automated cell counter (PocH – 100iv Diff). Red blood cell morphology and leukocyte differentiation (neutrophils, lymphocytes, eosinophils, monocytes, and basophils) were assessed via Rosenfeld-stained blood smears. All analyses were completed within 30 min of sample collection.

Serum biochemical parameters were measured using an automatic spectrophotometer (LABTEST, LabQuest, São Paulo, Brazil) with commercial assay kits, according to the manufacturer’s recommendations. Analytes included creatinine (mg/dL), urea (mg/dL), total protein (TP) (g/dL), albumin (g/dL), globulins (g/dL), total bilirubin (TB) (mg/dL), direct bilirubin (DB) (mg/dL), indirect bilirubin (IB) (mg/dL), alanine aminotransferase (ALT) (U/L), alkaline phosphatase (ALP) (U/L), glucose (mg/dL), total calcium (tCa) (mg/dL), phosphorus (P) (mg/dL), creatine kinase (CK) (U/L), and lactate (mmol/L).

Urine samples were centrifuged immediately after collection, aliquoted, and stored in liquid nitrogen to preserve protein stability until analysis. Serum samples were processed under the same conditions. Serum symmetric dimethylarginine (SDMA) concentrations were measured using a canine-specific ELISA kit (Bioassay Technology Laboratory, Shanghai, China). Urinary TIMP-2 and IGFBP7 concentrations were determined using canine-specific sandwich ELISA kits from the same manufacturer. Urinary biomarkers were evaluated both as absolute concentrations and after normalization to urinary creatinine (TIMP-2/uCr and IGFBP7/uCr).

### 2.5 Preoperative, surgical, anesthetic, and postoperative procedures

None of the dogs received medical treatment prior to hospital admission. After samples collection, all animals returned to the hospital routine for medical/surgical treatment, according to the institutional protocols and underwent ovariohysterectomy under a standardized anesthetic protocol to minimize variability. Premedication consisted of intramuscular methadone (0.3 mg/kg), followed by intravenous induction with propofol (5 mg/kg) and orotracheal intubation. Anesthesia was maintained with isoflurane and fentanyl (0.005 mg/kg). Once the ovariohysterectomy was over, a biopsy of the right renal cortex was collected with a semiautomatic biopsy needle (TRU-CUT, Velox, São Paulo, Brazil) (14G or 16G, according to patient size), following the protocol described by Crivellenti et al. (24). After fragment collection, local compression was applied for at least 3 min to ensure hemostasis. Postoperative analgesia and antibiotic therapy were administered uniformly to all patients.

Dogs were monitored postoperatively through ultrasonographic evaluation and hematocrit measurement at 6-h intervals during the first 24 h to detect potential biopsy-related complications.

### 2.6 Histopathological evaluation

Biopsy samples were immediately fixed in alcoholic Bouin’s solution for at least 5 h, followed by routine histological processing and paraffin embedding. Sections of 2–3 μm were stained with hematoxylin and eosin (HE), periodic acid–Schiff (PAS), Masson’s trichrome and Jones methenamine silver to assess tubular, glomerular and interstitial alterations. Histopathological grading of renal injury severity was based on semiquantitative evaluation using a 0–3 scale (0 = absent, 1 = discrete, 2 = moderate, 3 = severe). Microscopic assessment was performed by two experienced veterinary pathologists blinded to the clinical and laboratory status of the animals, and final lesion classification was determined by consensus interpretation, following the standards of the International Veterinary Renal Pathology Service (IVRPS).

### 2.7 Interpretation of slides

Pathological analyses included the evaluation of tubular injury (TI), fibrosis and tubular atrophy (FIAT), interstitial inflammatory infiltrate (INFInf), periglomerular infiltrate (InfPG), membranoproliferative glomerulonephritis (MPGN), and glomerular sclerosis (Esc).

Tubular injury (TI) was assessed based on characteristic features of epithelial degeneration, including hydropic changes (apical blebs, cellular swelling and vacuolization), flattening or loss of brush border, reduced epithelial height, detachment from the basement membrane, basal membrane irregularity, and tubular dilation. Hallmarks of acute tubular necrosis (ATN), such as necrotic epithelial cells and tubular cell casts, were also considered. TI severity was graded using a 0–3 scale:

0 = absent.

1 = discrete (scattered injured tubules).

2 = moderate (multifocal lesions with cellular detachment).

3 = severe (extensive necrosis involving most of the cortical tubules).

FIAT was defined by reduced tubular density and diameter, with interstitial expansion due to fibrosis or parenchymal loss.

INFInf was classified according to the extent of inflammatory cell infiltrates in the interstitium.

Both FIAT and INFInf were initially estimated as the percentage of affected parenchyma and subsequently categorized using the same 0–3 scale:

0 = ≤5% involvement.

1 = 6–25% (discrete).

2 = 26–50% (moderate).

3 = > 50% (diffuse).

Glomerular lesions, including InfPG, MPGN and sclerosis, were evaluated based on the proportion of affected glomeruli. MPGN was identified by mesangial hypercellularity, expansion of mesangial matrix and basement membrane thickening. Glomerular sclerosis was diagnosed by widespread hyalinization with loss of capillary lumen definition. These lesions were graded on the same 0–3 scale based on the number of glomeruli involved:

0 = no involvement.

1 = 6–25% (discrete).

2 = 26–50% (moderate).

3 = > 50% (diffuse).

### 2.8 Experimental groups

Two histopathological classification systems were applied to assess the severity of renal injury:

1. Overall Lesion Severity Classification

Dogs were categorized based on the cumulative renal injury score:

- Discrete Group (D): total lesion score ≤ 2
- Moderate Group (M): total lesion score 3–4
- Severe Group (S): total lesion score ≥ 5

1. Tubular Lesion Grade Classification

Animals were further classified according to the degree of tubular degeneration:

- Grade 0 (Control, C): absence of tubular lesions
- Grade 1: discrete tubular degeneration
- Grade 2: moderate tubular degeneration
- Grade 3: severe tubular degeneration

## 3 Statistical analysis

Statistical analyses were performed using R software (version 4.1.1). Continuous data were expressed as median and interquartile range (IQR). Comparisons among the discrete, moderate, and severe groups were performed using the Kruskal–Wallis test. When a statistically significant overall difference was detected, pairwise comparisons were conducted using Dunn’s post-hoc test. Statistical significance was defined as *p* < *0.05*.

Principal component analysis (PCA) was performed using the FactoMineR package to explore multivariate patterns and identify variables contributing most to intergroup variability. PCA was applied separately according to (1) overall histopathological lesion severity classification (discrete, moderate, and severe) and (2) tubular lesion grade classification (grades 0, 1, 2, and 3). The contribution of each variable was visualized using biplots.

### 3.1 Results

#### 3.1.1 Experimental groups and histopathological evaluation

**Figure 1:**
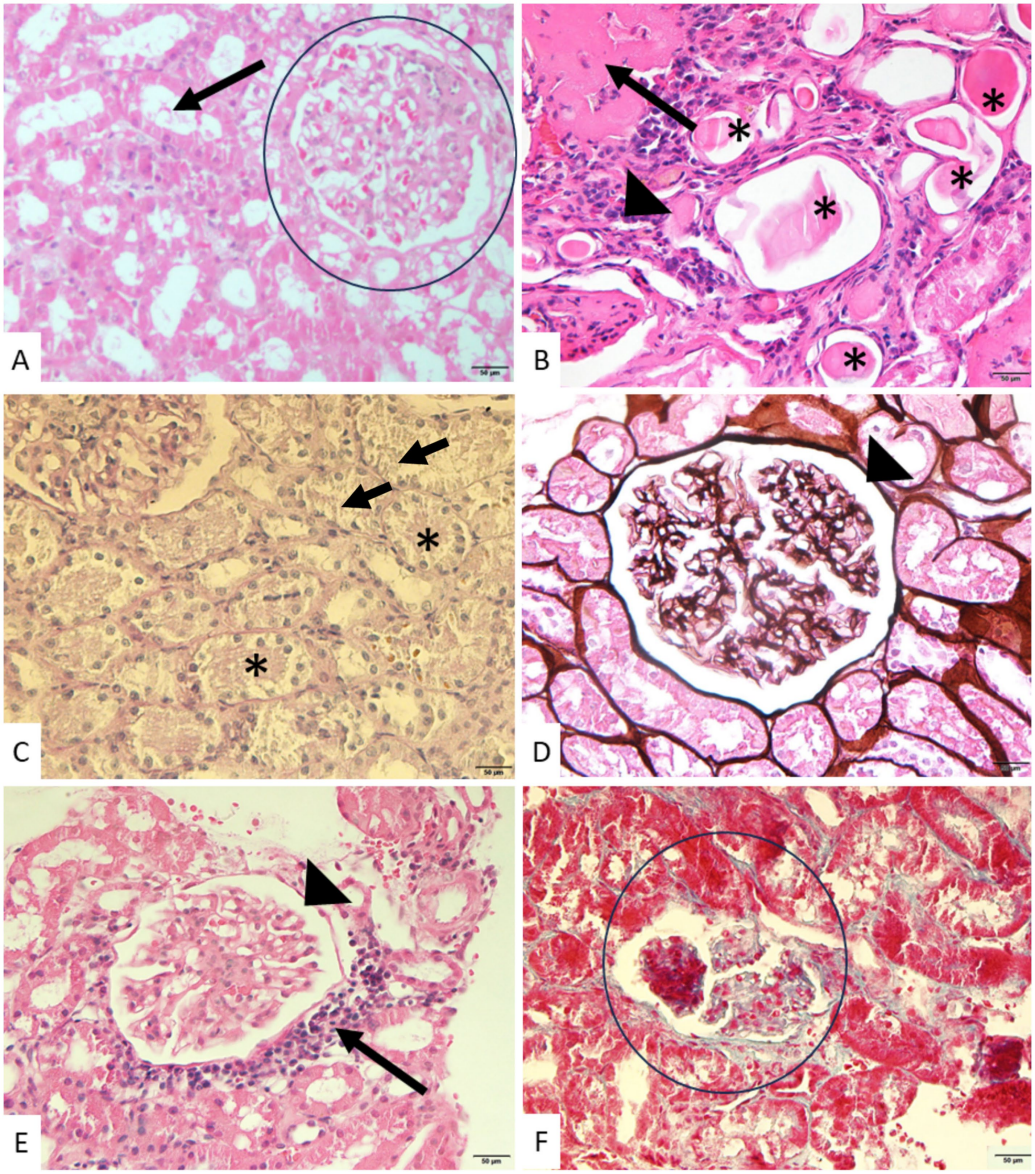
Photomicrographs of kidneys from bitches with pyometra. **(A)** Normal glomerular (circle) and tubular (arrow) architecture, showing preserved epithelial cell morphology and absence of significant inflammatory or degenerative changes. HE. Scale bar: 50 μm. **(B)** Tubules with a large amount of hyaline casts (asterisks), interstitial inflammatory infiltrate (arrowhead), and tubular atrophy (black arrow). HE. Scale bar: 50 μm. **(C)** Grade 1 tubular epithelial degeneration showing marked attenuation of the PAS-positive brush border (arrows) and cytoplasmic vacuolar degeneration. Cellular debris is present within the tubular lumen (asterisks). PAS. Scale bar: 50 μm. **(D)** Grade 1 membranoproliferative glomerulonephritis. Thickening of the glomerular basement membrane (arrowhead). Jones methenamine silver stain. Scale bar: 50 μm. **(E)** Grade 2 membranoproliferative glomerulonephritis. Thickening of the glomerular basement membrane (arrowhead) and periglomerular inflammatory infiltrate (arrow). HE. Scale bar: 50 μm. **(F)** Severe tubular injury (Grade 3) with marked epithelial degeneration and architectural disruption surrounding the glomerulus (circle). Masson’s trichrome. Scale bar: 20 μm. Microscope: Olympus BX60.

Histopathological analysis confirmed that all dogs with pyometra (27/27; 100%) presented renal lesions of varying severity. Tubular injury was the most prevalent finding, observed in 85% (23/27) of cases, and classified as discrete in 48% (11/23), moderate in 35% (8/23) and severe in 17% (4/23). In two specimens, tubular thyroidization and intraluminal hyaline material were also observed (Figures 1A,B).

Fibrosis and tubular atrophy (FIAT) were detected in 48% (13/27) of samples, with discrete lesions in 54% (7/13) and moderate in 46% (6/13) (Figure 1B). Interstitial inflammatory infiltrates were present in 67% (18/27), predominantly discrete (67%; 12/18), followed by moderate (28%; 5/18) and severe (5%; 1/18) grades (Figures 1B,C).

Periglomerular infiltrate was identified in 52% (14/27) of animals, with the majority classified as discrete (79%; 11/14), moderate in 14% (2/14) and severe in 7% (1/14). Membranoproliferative glomerulonephritis (MPGN) was detected in 63% (17/27) of samples, evenly distributed between discrete (35%; 6/17) and moderate (35%; 6/17) lesions, and severe in 30% (5/17) (Figures 1D,E). Membranous glomerulonephritis (MGN) was not identified in any sample. Renal sclerosis was observed in 37% (10/27) of cases, mostly discrete (80%; 8/10), with one moderate (10%; 1/10) and one severe (10%; 1/10) finding (Figure 1F).

Based on the cumulative histopathological lesion scores, animals were stratified into three groups: discrete (*n* = 8), moderate (*n* = 8) and severe (*n* = 11). All patients exhibited at least one type of renal injury. Detailed individual histological scoring is available in Supplementary Table 1.

#### 3.1.2 Clinical parameters

On physical examination, most patients exhibited systemic clinical alterations, with apathy recorded in 89% (24/27) of cases. Reduced food intake was frequently reported, including hyporexia in 65% (13/27) and anorexia in 74% (20/27) of animals. Gastrointestinal signs were also common, with vomiting observed in 52% (14/27) and diarrhea in 41% (11/27).

Regarding urine output, 56% (15/27) of dogs showed no change, whereas 26% (7/27) exhibited polyuria and 19% (5/27) presented oliguria.

Reproductive history revealed that 56% (15/27) of the animals had a recent estrous cycle, defined as occurring within up to 12 weeks before hospital admission. Additionally, 37% (10/27) had a history of pseudopregnancy, and 40% (8/27) had previously received progestogen treatment. Vaginal discharge was present in 48% (13/27) of dogs.

No significant differences were identified among the discrete, moderate and severe renal lesion groups for any of the clinical parameters evaluated.

#### 3.1.3 Complete blood count

Hematological parameters did not differ significantly among the discrete, moderate and severe renal lesion groups (*p >* 0.05; Supplementary Table 2).

#### 3.1.4 Biochemical analysis

Most biochemical variables were similar across groups (*p > 0.05*; Supplementary Table 3).

However, blood urea nitrogen (BUN) was significantly higher in animals classified as severe (median 36.00; IQR 27.90 mg/dL) compared with those in the discrete group (median 20.25; IQR 9.15 mg/dL; *p* = 0.0358). No other statistically significant differences were observed.

#### 3.1.5 Urinalysis and blood gas analysis

No significant differences (*p > 0.10*) were observed among groups for urinalysis or blood gas parameters (Supplementary Table 4).

#### 3.1.6 Renal biomarkers

**Figure 2:**
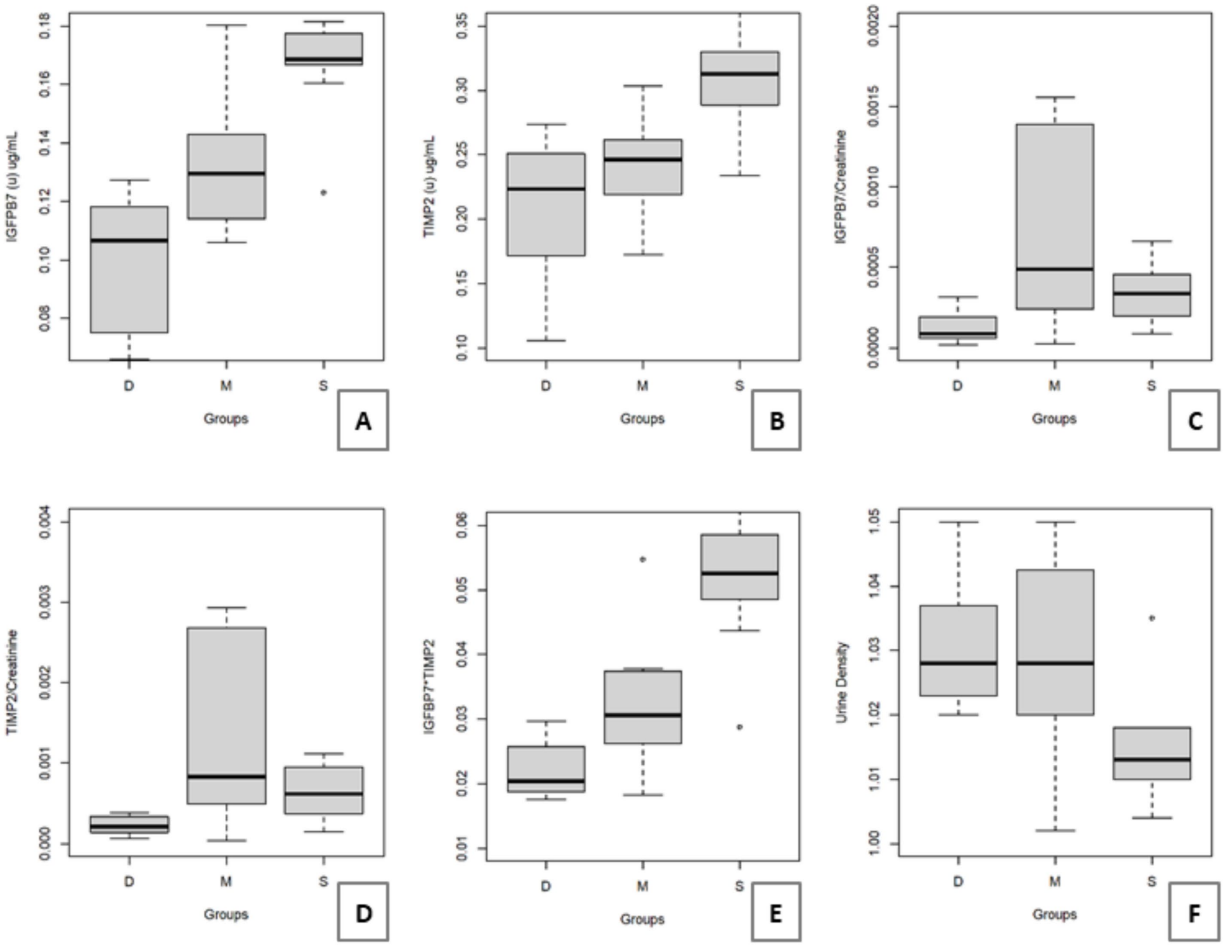
Box plots comparing urinary biomarkers and urine specific gravity among groups (discrete, moderate, and severe). **(A)** Urinary IGFBP7 (μg/mL) was significantly higher in the severe group compared with the moderate group, while the discrete group showed lower values than both groups (*p < 0.05*). **(B)** Urinary TIMP-2 (μg/mL) was significantly higher in the severe group compared with the discrete and moderate groups (*p = 0.0006*). **(C)** The urinary IGFBP7/creatinine ratio showed significantly higher values in the moderate and severe groups compared with the discrete group (*p = 0.0189*). **(D)** The urinary TIMP-2/creatinine ratio was significantly higher in the moderate and severe groups compared with the discrete group (*p = 0.0153*). **(E)** The combined index [IGFBP7] × [TIMP-2] was significantly higher in the severe group compared with the moderate and discrete groups, and values in the moderate group were also higher than those in the discrete group (*p = 0.0001*). **(F)** Urine specific gravity was significantly lower in the severe group compared with the discrete and moderate groups (*p = 0.0022*).

No significant differences were observed in conventional renal biomarkers, including Cr(s), SDMA(s) and UPC, across the discrete, moderate and severe groups (*p* > 0.10; Table 1). Urinary IGFBP7 concentrations were significantly higher in animals classified as severe compared with the moderate group (*p* < 0.05), while the discrete group showed the lowest values (Figure 2A). Urinary TIMP-2 was also significantly elevated in the severe group compared with both discrete and moderate groups (*p* = 0.0006; Figure 2B).

**Table 1:** Median ± interquartile range of biomarkers in female dogs diagnosed with pyometra, grouped by the sum of histopathology lesion scores into categories: discrete (equal to or less than 2), moderate (equal to 3 or 4), and severe (equal to or greater than 5).

	Groups			*p*-value
	Discrete	Moderate	Severe	
Creatinine (mg/dL)	1,00 ± 0,44	0,76 ± 0,25	0,80 ± 0,34	0,3,672
Urea (mg/dL)	24,50 ± 13,12	31,50 ± 12,25	24,20 ± 23,05	0,7,272
DU	1,028 ± 0,004^a^	1,028 ± 0,027^a^	1,012 ± 0,008^b^	0,0022
UPC	0,13 ± 0,43	0,18 ± 0,30	0,50 ± 0,51	0,1,119
GGT(u) UI/L	74,50 ± 257,75	81,90 ± 206,10	64,40 ± 76,20	0,2,820
GGT(u)/Creat (u)	0,731 ± 0,387^b^	6,373 ± 10,740^a^	0,747 ± 2,095^b^	0,0059
SDMA(s) μg/mL	0,24 ± 0,03	0,17 ± 0,11	0,23 ± 0,08	0,1819
IGFBP7(u) μg/mL	0,106 ± 0,04^c^	0,129 ± 0,02^b^	0,168 ± 0,01^a^	0,0001
IGFBP7(u)/Cr(u), n 10^−5^	8,56 ± 12,39^b^	48,83 ± 104,68^a^	33,71 ± 24,37^a^	0,0189
TIMP-2(u) μg/mL	0,22 ± 0,06^b^	0,24 ± 0,03^b^	0,31 ± 0,04^a^	0,0006
TIMP-2(u)/Cr(u), n 10^−4^	4,88 ± 1,64^b^	8,28 ± 20,32^a^	6,20 ± 5,10^a^	0,0153
[IGFBP7].[TIMP-2]	0,020 ± 0,005^c^	0,030 ± 0,009^b^	0,052 ± 0,009^a^	0,0001

IGFBP7 = insulin-like growth factor binding protein 7. TIMP-2 = tissue inhibitor of metalloproteinases 2. GGT = gamma-glutamyltransferase. SDMA = symmetric dimethylarginine. Lowercase superscript letters on the same line indicate statistically significant differences according to the Kruskal–Wallis test followed by Dunn’s post-hoc test.

When normalized to urinary creatinine, both IGFBP7(u)/Cr(u) and TIMP-2(u)/Cr(u) were significantly higher in the moderate and severe groups compared with the discrete group (*p* = 0.0189 and *p* = 0.0153, respectively; Figures 2C,D). The combined index [IGFBP7] × [TIMP-2] showed a progressive increase consistent with histopathological severity (*p* = 0.0001; Figure 2E).

Urine density was significantly lower in the severe group (median 1.010; IQR 0.01) compared with the discrete and moderate groups (*p* = 0.0022; Figure 2F).

#### 3.1.7 Principal component analysis based on cumulative lesion scores

**Figure 3:**
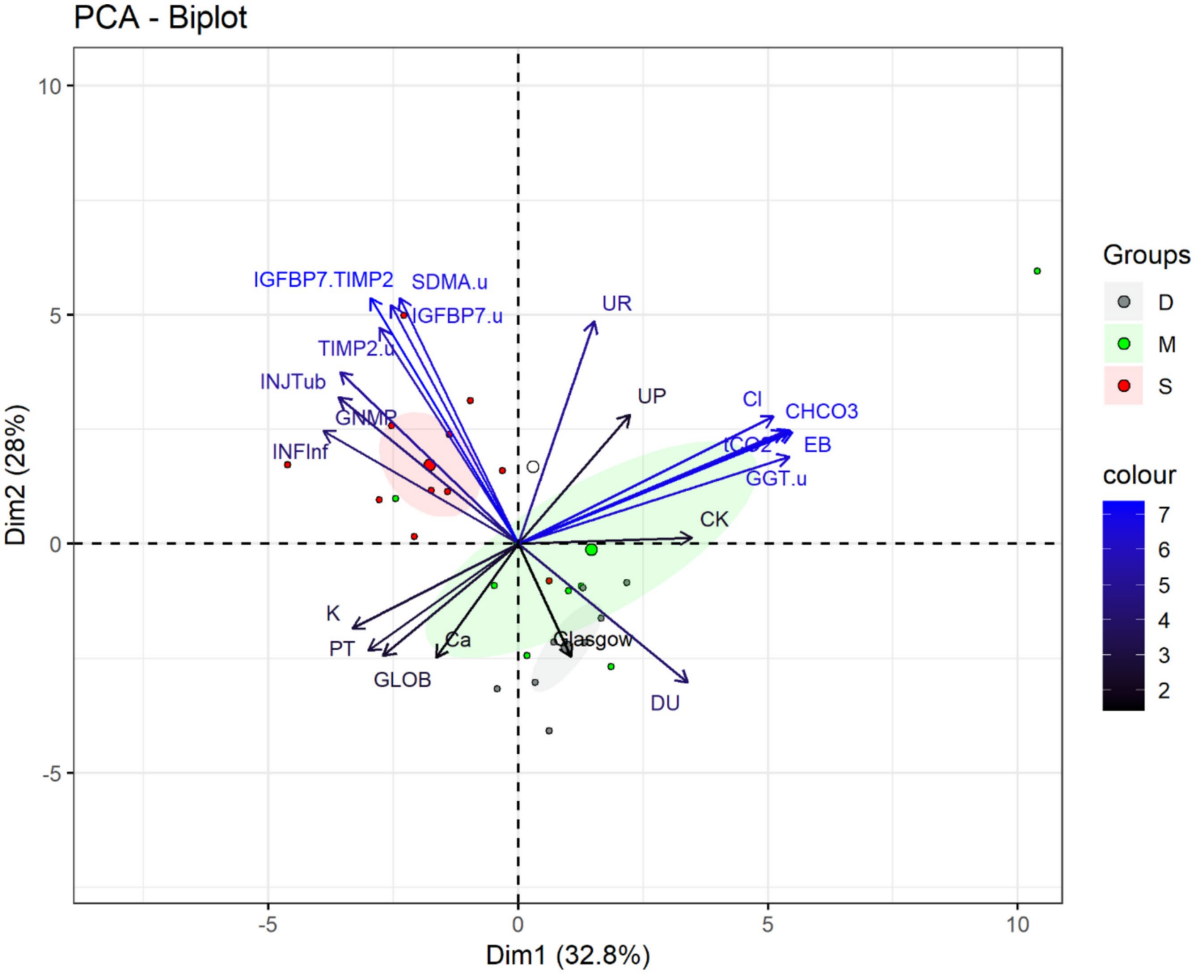
Biplot of principal component analysis (PCA) of explanatory variables for the separation of female dogs diagnosed with pyometra, grouped by the sum of histopathology lesion scores into discrete (≤2), moderate (=3 or 4), and severe (≥5) groups. Variables with the greatest contribution are shown in blue. Larger circles at the center of the ellipses represent the mean of animals in each group. INFinf = Interstitial inflammatory infiltrate. GNMp = Membranoproliferative glomerulonephritis. INJTub = Tubular injury. TIMP-2 = Tissue inhibitor of metalloproteinases 2 (urinary) or TIMP-2(u). IGFBP7.u = Insulin-like growth factor binding protein 7 (urinary) or IGFBP7(u). TIMP2.u = Tissue inhibitor of metalloproteinases 2. IGFBP7. TIMP-2 = IGFBP7 index associated with TIMP-2 or [IGFBP7].[TIMP-2]. GGT.u = Urinary gamma-glutamyltransferase or GGT(u). SDMA.u = Urinary symmetric dimethylarginine. Cl = Chloride (Cl^−^). CHCO_3_ = Bicarbonate. UR = Urea. tCO_2_ = Total carbon dioxide concentration. PT = Total protein. K = Potassium. Ca = Ionic calcium or Ca^+2^. CK = Creatine kinase. EB = Base excess. SDMA(u) appears in the PCA output as part of the statistical dataset but was not addressed in the present manuscript.

Principal Component Analysis (PCA) identified 21 out of the 107 evaluated variables, which together explained 60.8 percent of the variance and effectively differentiated the severe group from the discrete and moderate groups (Figure 3). Dogs classified as severe were primarily located in the negative region of component 1 (Dim1) and the positive region of component 2 (Dim2), whereas animals in the discrete and moderate groups clustered mainly in the negative region of Dim2.

The severe group showed strong positive associations with urinary biomarkers and histopathological parameters, including [IGFBP7] × [TIMP-2] (*r* = 0.85), IGFBP7(u) (*r* = 0.82), TIMP-2(u) (*r* = 0.75), tubular degeneration (INJTub; *r* = 0.59), membranoproliferative glomerulonephritis (GNMP; *r* = 0.57), and interstitial inflammation (INFInf; *r* = 0.62). These variables were negatively correlated with urine density (*r* = −0.54), indicating a relationship between increased biomarker expression, structural renal injury and reduced urine concentration ability and clinical deterioration.

In contrast, variables indicative of physiological stability and metabolic compensation, including base excess (BE; *r* = 0.87), bicarbonate (cHCO₃; *r* = 0.86), total CO₂ (tCO₂; *r* = 0.84), chloride (Cl^−^; *r* = 0.81), GGT(u) (*r* = 0.86), pH (*r* = 0.72), creatine kinase (CK; *r* = 0.55), and urine density (DU; *r* = 0.54), were positively associated with discrete and moderate groups. These variables were negatively correlated with transitional epithelial cells (*r* = −0.56), potassium (*r* = −0.53), total protein (*r* = −0.47), globulins (*r* = −0.43), and ionized calcium (*r* = −0.40).

Serum urea (*r* = 0.77) did not show a specific cluster association, suggesting limited discriminatory ability, although its inverse correlation with K^+^, PT, Ca^2+^, and globulins may reflect systemic metabolic changes rather than direct renal damage.

#### 3.1.8 Principal component analysis based on degrees of tubular degeneration

**Figure 4:**
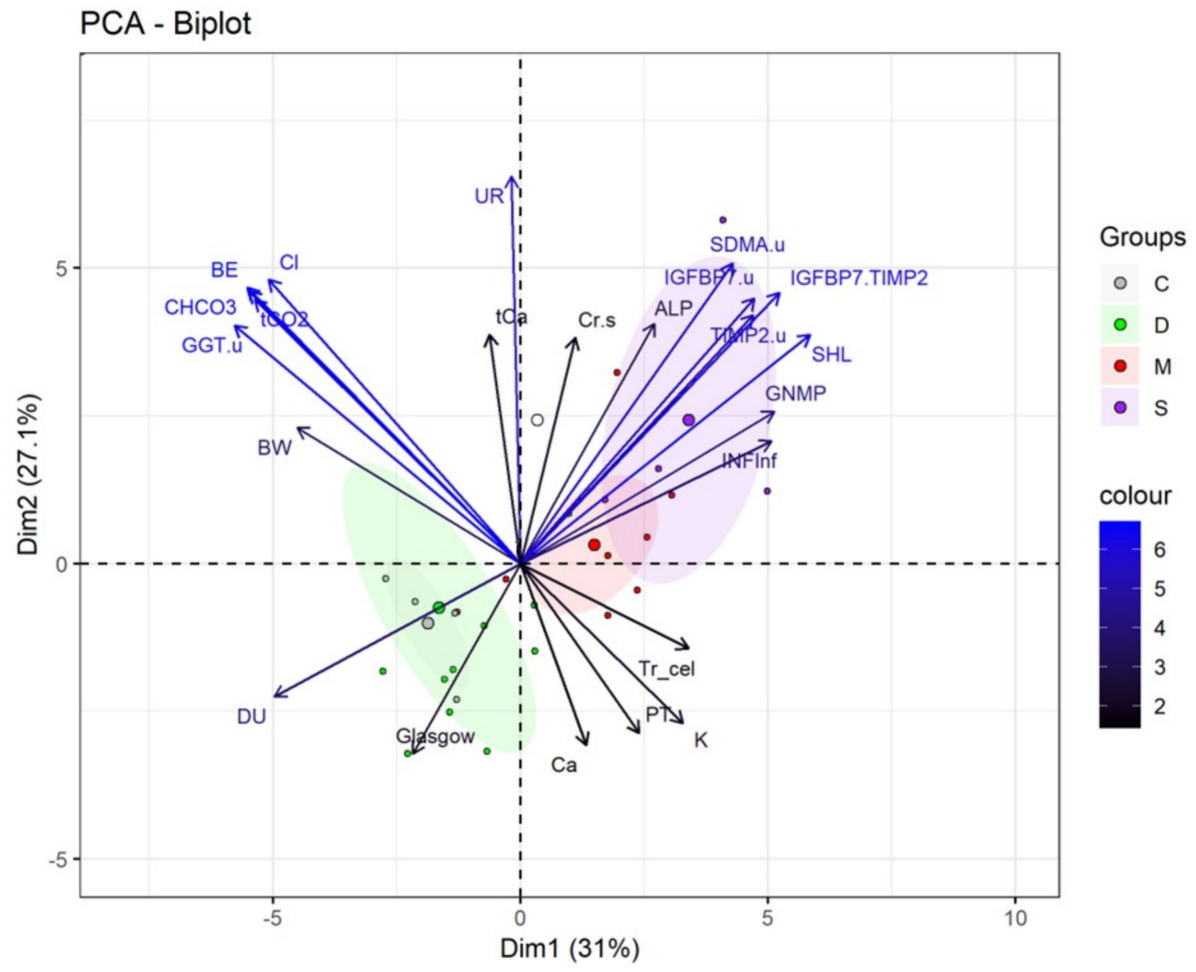
Biplot of principal component analysis (PCA) of explanatory variables for the separation of female dogs diagnosed with pyometra, grouped by histopathology lesion score of tubular degeneration into control (C), discrete (D), moderate (M), and severe (S) groups. Variables with the greatest contribution are shown in blue. Larger circles at the center of the ellipses represent the mean of the animals in each group. INFinf = Interstitial inflammatory infiltrate. GNMp = Membranoproliferative glomerulonephritis. INJTub = Tubular injury. TIMP-2 = Tissue inhibitor of metalloproteinases 2 (urinary) or TIMP-2(u). IGFBP7.u = Insulin-like growth factor binding protein 7 (urinary) or IGFBP7(u). TIMP2.u = Tissue inhibitor of metalloproteinases 2. IGFBP7. TIMP-2 = IGFBP7 index associated with TIMP-2 or [IGFBP7].[TIMP-2]. GGT.u = Urinary gamma-glutamyltransferase or GGT(u). Cr.s = Serum creatinine or Cr(s). Cl = Chloride or Cl^−^. CHCO_3_ = Bicarbonate or cHCO_3_. UR = Urea. tCO_2_ = Total carbon dioxide concentration. PT = Total protein. K = Potassium. UI. L = International units per liter. Ca = Ionic calcium or Ca^+2^. CK = Creatine kinase. EB = Base excess. BW = Body weight. tCa = Total calcium. DU = Urine density. SHL = Sum of histopathology lesions. TR_Cel = Transitional cells. ALP = Alkaline phosphatase or FA. SDMA(u) appears in the PCA output as part of the statistical dataset but was not addressed in the present manuscript.

Principal Component Analysis (PCA) identified 24 out of 107 evaluated variables that together explained 58.1 percent of the accumulated variance among groups classified according to tubular degeneration severity (Figure 4). Animals in the moderate and severe groups were primarily located in the positive region of component 1 (Dim1), whereas those in the control and discrete groups were distributed in the negative region of Dim1.

The moderate and severe groups showed positive associations with cumulative histopathological lesion score (SHL; *r* = 0.78), the combined biomarker [IGFBP7] × [TIMP-2] (*r* = 0.69), membranoproliferative glomerulonephritis (MPGN; *r* = 0.68), interstitial inflammation (INFInf; *r* = 0.67), IGFBP7(u) (*r* = 0.63), TIMP-2(u) (*r* = 0.62), and tubular degeneration (INJTub; *r* = 0.54).

Conversely, control and discrete groups were predominantly associated with urine density (DU; *r* = −0.66), body weight (*r* = −0.60), reflecting greater physiological compensationy.

Serum urea (*r* = 0.87), total calcium (*r* = 0.51), and serum creatinine [Cr(s); *r* = 0.51] were more closely positioned toward animals with more marked tubular lesions, although without exclusive clustering in any group.

Variables indicative of metabolic stability and fluid-electrolyte regulation, including base excess (BE; *r* = −0.73), bicarbonate (cHCO₃; *r* = −0.73), total CO₂ (tCO₂; *r* = −0.71), chloride (Cl^−^; *r* = −0.67), and GGT(u) (*r* = −0.77), showed inverse distribution relative to ionized calcium (*r* = −0.41), potassium (*r* = 0.43), transitional epithelial cells (*r* = 0.45), and total protein (*r* = −0.38).

## 4 Discussion

The present study investigated the occurrence and severity of renal lesions in bitches with pyometra under systemic inflammatory response and evaluated whether urinary IGFBP7 and TIMP-2 could reflect histopathological renal alterations more accurately than conventional renal biomarkers. Our findings support the proposed hypothesis, as urinary IGFBP7, TIMP-2, and their combined index demonstrated progressive associations with tubular, interstitial, and glomerular injuries, whereas serum Cr(s), SDMA(s), and UPC did not discriminate among severity groups.

Histopathological analysis showed that all animals, although non-azotemic, exhibited some degree of renal injury. Tubular injury was the most prevalent lesion, corroborating previous reports indicating that tubular epithelial cells are among the earliest and most affected structures in inflammatory and ischemic conditions (3, 16), likely due to their high metabolic demand and susceptibility to hypoxic and oxidative stress during systemic inflammation (25, 26). Most lesions were graded as discrete or moderate, characterized by cellular swelling, brush border loss, and focal detachment. Few animals exhibited grade 3 lesions consistent with necrosis, which is similar to findings in studies describing naturally occurring inflammatory renal injury as often focal and heterogeneous (27, 28).

Interstitial inflammatory infiltrates and glomerular alterations, including membranoproliferative glomerulonephritis and periglomerular infiltrate, were observed in over half of the animals. These findings suggest that tubular injury alone does not fully account for renal impairment. Previous studies have indicated that inflammatory and microvascular disturbances may extend beyond tubular structures, contributing to glomerular and interstitial damage (29, 30). Such structural complexity may partly explain why biomarkers reflecting cellular stress, rather than solely glomerular filtration, demonstrated stronger associations with lesion severity in the present study. However, UPC did not differ significantly among groups, indicating that structural glomerular changes did not result in clinically detectable proteinuria. This supports previous observations that proteinuria may be absent in early inflammatory AKI when glomerular permeability is not extensively compromised (3).

Urinary TIMP-2 and IGFBP7 demonstrated clearer associations with renal injury severity than serum markers. Levels of both biomarkers, especially when normalized to urinary creatinine, progressively increased from the discrete to the severe lesion groups. TIMP-2(u) and IGFBP7(u), alone and in combination, showed significant stratification of renal injury grades, including differences between moderate and severe lesions. These findings align with studies in human medicine, where IGFBP7 and TIMP-2 are released by stressed tubular cells during G1 cell cycle arrest to prevent cell division under injury conditions (18, 21). Their urinary excretion reflects tubular cellular stress rather than merely functional decline, making them suitable early markers of non-azotemic AKI (17).

In veterinary medicine, research on cell-cycle arrest biomarkers is still limited, with only a few recent studies evaluating their diagnostic potential. Biscop et al. (22) demonstrated that uTIMP-2/uCr and [TIMP-2] × [IGFBP7]/uCr were significantly increased in dogs with AKI compared to animals with chronic kidney disease, heart failure or healthy controls. Dorn et al. (23) further reported that urinary TIMP-2, particularly when normalized to creatinine, was higher in non-surviving critically ill dogs with AKI, supporting its association with severity and prognosis. While previous studies assessed biomarker performance without histopathological correlation, their findings support the clinical relevance of urinary TIMP-2 and IGFBP7. The present study extends these observations by demonstrating an association between urinary cell-cycle arrest biomarkers and histopathologically confirmed renal lesion severity in clinically non-azotemic dogs.

In contrast, serum SDMA, Cr(s), and UPC failed to differentiate among histopathological groups. These markers are known to reflect functional impairment rather than early cellular damage (12, 13). Their lack of discriminatory ability in the present study reinforces the concept that structural renal injury may precede measurable functional impairment, particularly in inflammatory conditions. The absence of significant variation in Cr(s) or SDMA(s) therefore supports the occurrence of renal injury without overt declines in glomerular filtration, highlighting the relevance of subclinical AKI (31).

Principal component analyses further supported these observations. Variables most strongly associated with moderate and severe renal injuries included IGFBP7(u), TIMP-2(u), the combined index [IGFBP7] × [TIMP-2], and histopathological scores, particularly tubular degeneration and interstitial inflammation. Conversely, urine specific gravity and body weight were associated with control and discrete groups, suggesting preserved renal concentrating ability in milder injury stages.

Although clinical, ultrasonographic and laboratory examinations did not indicate chronic kidney disease, some animals presented focal histopathological features such as glomerular sclerosis or interstitial fibrosis. These alterations were mild, not predominant and did not correlate with biomarker levels, suggesting that they likely reflected incidental age-related microscopic changes rather than clinically relevant chronic processes. Similar findings have been reported in healthy adult dogs (32).

Among biochemical parameters, urea was significantly higher in the severe group but remained within near-normal range. This increase may reflect mild renal dysfunction, dehydration or protein catabolism, but its diagnostic specificity remains limited (33). No consistent correlations were observed between urea and TIMP-2 or IGFBP7, confirming that these biomarkers are not surrogates of nitrogenous waste accumulation, but of cellular injury.

This study presents some limitations. The absence of a negative control group without renal injury restricted comparisons. Additionally, the lack of serial biomarker measurements prevented inferences regarding temporal dynamics. Furthermore, the cross-sectional design precludes conclusions about the temporal onset, duration, or progression of biomarker elevation. As this study included naturally occurring clinical cases, the exact duration of pyometra and potential renal injury prior to presentation could not be established. It was not possible to determine cutoff values or sensitivity and specificity, as that was beyond the scope of this exploratory study. Future studies should incorporate longitudinal designs, larger sample sizes, and integration of molecular techniques to evaluate biomarker performance at cellular and transcriptomic levels.

In summary, urinary IGFBP7 and TIMP-2 were associated with histopathologically confirmed renal lesions in the pre-azotemic stage. Their secretion appeared closely related to renal histopathological severity and showed improved performance compared with conventional markers in this context. These findings suggest that these biomarkers may be potentially useful for risk assessment or monitoring of renal injury. Although the present findings were obtained in animals experiencing systemic inflammation, further studies are warranted to determine their applicability in other clinical conditions.

## Data Availability

The original contributions presented in the study are included in the article/Supplementary material, further inquiries can be directed to the corresponding author.
