# Supplementary material for: Urinary cell cycle arrest biomarkers TIMP-2 and IGFBP7 for the assessment of acute kidney injury in dogs with pyometra
Source: Front Vet Sci. 2026 Mar 10;13:1788906. doi: 10.3389/fvets.2026.1788906 (PMC13008719; doi:10.3389/fvets.2026.1788906)
Supplement: Supplementary file 1 [file Table_1.DOCX]

Supplementary Material

Urinary cell cycle arrest biomarkers TIMP-2 and IGFBP7 for the assessment of acute kidney injury in dogs with pyometra

Larissa A. do N. Braz1*, Suellen R. Maia2, Beatriz Gasser3, Nathan da R. N. Cruz4, Larissa F. Magalhães5, Lara Vilela Soares7, Ricardo A. R. Uscategui6, Andrigo B. de Nardi1, Leandro Z. Crivellenti7

*** Correspondence:** larissa.ayane@unesp.br

# Supplementary Figures and Tables

Table 1. Histopathological data results from the experiment on dogs with pyometra scored according to the severity of each lesion, in accordance with the "International Veterinary Renal Pathology Service" (IVRPS).

| Number | INJTub | FIAT | INFInf | InfPGl | MGN | MPGN | Esc | Som | Grupo |
| --- | --- | --- | --- | --- | --- | --- | --- | --- | --- |
| 1 | 1 | 0 | 1 | 0 | 0 | 0 | 0 | 2 | D |
| 2 | 2 | 1 | 0 | 0 | 0 | 0 | 0 | 3 | M |
| 3 | 2 | 2 | 0 | 0 | 0 | 2 | 0 | 6 | S |
| 4 | 1 | 0 | 1 | 0 | 0 | 0 | 0 | 2 | D |
| 5 | 1 | 0 | 1 | 0 | 0 | 2 | 0 | 4 | M |
| 6 | 0 | 0 | 0 | 1 | 0 | 0 | 1 | 2 | D |
| 7 | 3 | 2 | 2 | 0 | 0 | 3 | 3 | 13 | S |
| 8 | 2 | 0 | 0 | 0 | 0 | 3 | 1 | 6 | S |
| 9 | 1 | 1 | 0 | 1 | 0 | 0 | 0 | 3 | M |
| 10 | 2 | 2 | 2 | 2 | 0 | 2 | 1 | 11 | S |
| 11 | 2 | 2 | 2 | 1 | 0 | 1 | 1 | 9 | S |
| 12 | 2 | 0 | 1 | 0 | 0 | 0 | 2 | 4 | M |
| 13 | 1 | 0 | 1 | 1 | 0 | 1 | 0 | 4 | M |
| 14 | 1 | 1 | 0 | 0 | 0 | 1 | 1 | 4 | M |
| 15 | 2 | 2 | 2 | 0 | 0 | 2 | 0 | 8 | S |
| 16 | 1 | 0 | 1 | 1 | 0 | 1 | 0 | 4 | M |
| 17 | 2 | 0 | 1 | 1 | 0 | 2 | 1 | 7 | S |
| 18 | 3 | 2 | 3 | 3 | 0 | 3 | 0 | 14 | S |
| 19 | 3 | 1 | 1 | 1 | 0 | 2 | 0 | 8 | S |
| 20 | 1 | 0 | 0 | 1 | 0 | 0 | 0 | 2 | D |
| 21 | 3 | 1 | 2 | 2 | 0 | 3 | 0 | 11 | S |
| 22 | 1 | 0 | 1 | 1 | 0 | 3 | 1 | 7 | S |
| 23 | 1 | 1 | 0 | 1 | 0 | 0 | 1 | 4 | M |
| 24 | 1 | 0 | 1 | 0 | 0 | 0 | 0 | 2 | D |
| 25 | 0 | 0 | 0 | 0 | 0 | 1 | 0 | 1 | D |
| 26 | 0 | 1 | 1 | 0 | 0 | 1 | 0 | 3 | D |
| 27 | 0 | 0 | 1 | 1 | 0 | 0 | 0 | 2 | D |

**Table 2.** Median ± interquartile range of blood count values in dogs diagnosed with pyometra grouped by the sum of the histopathology lesion scores into Discrete group (equal to or less than 2), Moderate group (equal to 3 or 4), and Severe group (equal to or greater than 5)

|  | **Groups** | | | **p-value** |
| --- | --- | --- | --- | --- |
|  | **Discret** | **Moderate** | **Severe** |  |
| Red Blood Cells, mm^3^ | 5.660.000 ± 2.705.000 | 5.750.000 ± 2.010.000 | 6.300.000 ± 1.500.000 | 0,5819 |
| Hemoglobin, g/dL | 13,50 ± 6,05 | 12,25 ± 5,67 | 13,40 ± 7,25 | 0,7756 |
| Hematocrit, % | 38,15 ± 26,90 | 36,10 ± 13,62 | 39,40 ± 9,35 | 0,5873 |
| MCV, µ | 65,90 ± 6,55 | 66,25 ± 4,95 | 62,20 ± 4,15 | 0,1453 |
| CHCM | 34,19 ± 0,99 | 35,30 ± 1,29 | 35,50 ± 1,95 | 0,3793 |
| Platelets, mm^3^ | 279.500 ± 224.750 | 342.000 ± 254.500 | 240.000 ± 18.700 | 0,5227 |
| Leukocytes, mm^3^ | 24.350 ± 19.450 | 24.450 ± 29.925 | 27.400 ± 35.000 | 0,9470 |
| Eosinophils, % | 2,49 ± 2,25 | 0,50 ± 2,25 | 0,00 ± 1,50 | 0,2017 |
| Eosinophils, mm^3^ | 800,5 ± 794,5 | 92,0 ± 493,5 | 0,00 ± 289,5 | 0,0890 |
| Band Cells% | 1,50 ± 3,75 | 3,00 ± 2,25 | 4,00 ± 3,50 | 0,2832 |
| Band Cells, mm^3^ | 207,50 ± 602,25 | 1285,50 ± 1.836 | 812,00 ± 2.021 | 0,1779 |
| Neutrophils% | 81,50 ± 10,25 | 76,50 ± 6,75 | 77,00 ± 11,00 | 0,3684 |
| Neut,rophils mm^3^ | 18.166 ± 8.460 | 19.761 ± 26.298 | 22.742 ± 28.241 | 0,9807 |
| Lymphocytes, % | 8,49 ± 7,00 | 13,50 ± 7,50 | 15,00 ± 6,50 | 0,0716 |
| Lyphocyte, mm^3^ | 2.658 ± 1.539 | 2.732 ± 929 | 4.032 ± 4.637 | 0,4313 |
| Monocytes, % | 1,50 ± 3,75 | 0,50 ± 4,25 | 1,00 ± 3,00 | 0,9466 |
| Monocytes, mm^3^ | 304,00 ± 873,00 | 106,50 ± 487,25 | 141,00 ± 1.107 | 0,7420 |
| Metamylocytes, mm^3^ | 0,00 ± 0,00 | 0,00 ± 0,00 | 0,00 ± 0,00 | 0,9530 |

MCV: Mean Corpuscular Volume; CHCM: Mean Corpuscular Hemoglobin Concentration. Lowercase superscript letters on the same line indicate significant differences according to the Kruskal-Wallis test and Dunn's post-test. No basophils were identified in the samples.

**Tabela 2.** Median ± interquartile range of biochemical values in dogs diagnosed with pyometra grouped by the sum of the histopathology lesion scores into Discrete group (equal to or less than 2), Moderate group (equal to 3 or 4), and Severe group (equal to or greater than 5).

|  | **Groups** | | | **p-valor** |
| --- | --- | --- | --- | --- |
|  | **Discrete** | **Moderate** | **Severe** |  |
| Creatinine, mg/dL | 0,91 ± 0,33 | 0,80 ± 0,35 | 0,90 ± 0,30 | 0,9283 |
| Urea, mg/dL | 20,25 ± 9,15^b^ | 24,00 ± 11,75^ab^ | 36,00 ± 27,90^a^ | 0,0358 |
| TP, g/dL | 7,75 ± 1,20 | 7,50 ± 2,87 | 7,50 ± 1,35 | 0,6871 |
| Albumin, g/dL | 2,27 ± 0,70 | 2,30 ± 0,52 | 2,40 ± 0,79 | 0,4094 |
| Globulins, g/dL | 5,83 ± 2,46 | 4,56 ± 2,75 | 4,60 ± 2,29 | 0,4724 |
| ALT, U/L | 21,00 ± 190,00 | 29,00 ± 34,67 | 27,00 ± 17,00 | 0,7848 |
| ALP, U/L | 184,00 ± 395,00 | 112,00 ± 98,55 | 250,00 ± 164,50 | 0,3744 |
| DB, mg/dL | 0,080 ± 0,20 | 0,105 ± 0,027 | 0,205 ± 0,197 | 0,3627 |
| TB, mg/dL | 0,205 ± 0,405 | 0,235 ± 0,167 | 0,320 ± 0,300 | 0,6073 |
| IB, mg/dL | 0,050 ± 0,202 | 0,135 ± 0,147 | 0,070 ± 0,447 | 0,6456 |
| CK, U/L | 185,00 ± 192,85 | 232,00 ± 240,50 | 90,80 ± 72,00 | 0,3483 |
| tCa, mg/dL | 9,20 ± 3,35 | 9,20 ± 1,62 | 10,70 ± 4,35 | 0,5230 |
| P, mg/dL | 6,30 ± 3,10 | 5,10 ± 0,87 | 5,75 ± 8,12 | 0,7380 |
| Lactate, mmol/L | 1,88 ± 0,11 | 2,11 ± 0,82 | 2,01 ± 1,51 | 0,7019 |
| Glucose, mg/dL | 88,50 ± 32,50 | 108,65 ± 11,12 | 93,00 ± 37,00 | 0,5386 |

Lowercase superscript letters on the same line indicate significant differences according to the Kruskal-Wallis test and Dunn's post-test. TP: total protein, g/dL; ALT: alanine aminotransferase, U/L; ALP: alkaline phosphatase, U/L; DB: direct bilirubin, mg/dL; TB: total bilirubin, mg/dL; IB: indirect bilirubin, mg/dL; CK: creatine kinase, U/L; tCa: total calcium, mg/dL; P: phosphorus, mg/dL.

**Tabela 3.** Median ± interquartile range of urinalysis and blood gas analysis values in dogs diagnosed with pyometra grouped by the sum of the histopathology lesion scores into Discrete group (equal to or less than 2), Moderate group (equal to 3 or 4), and Severe group (equal to or greater than 5).

|  | **Groups** | | | **p-valor** |
| --- | --- | --- | --- | --- |
|  | **Discrete** | **Moderade** | **Severe** |  |
| **Urinalysis** |  |  |  |  |
| Transitional cells | 1,00 ± 0,00 | 1,00 ± 1,25 | 1,00 ± 0,00 | 0,7634 |
| Renal cells | 0,14 ± 0,01 | 0,12 ± 0,00 | 0,10 ± 0,01 | 0,9649 |
| Hyalines casts | 0,00 ± 0,00 | 0,00 ± 0,01 | 0,00 ± 0,75 | 0,6044 |
| Granular casts | 0,57 ± 0,50 | 0,12 ± 0,00 | 0,10 ± 0,00 | 0,5177 |
| Bacteriuria | 1,00 ± 1,00 | 0,00 ± 1,00 | 1,00 ± 1,50 | 0,5099 |
| Hematuria | 1,00 ± 2,00 | 1,00 ± 0,50 | 1,00 ± 0,75 | 0,6642 |
| **Blood gas analysis** |  |  |  |  |
| pH | 7,42 ± 0,01 | 7,42 ± 0,04 | 7,43 ± 0,02 | 0,9138 |
| PCO_2,_ mmHg | 31,05 ± 3,82 | 31,05 ± 6,67 | 29,70 ± 5,30 | 0,6765 |
| PO_2,_ mmHg | 32,25 ± 71,15 | 36,10 ± 5,57 | 34,60 ± 11,70 | 0,6116 |
| cHCO_3 (mEq/L)_ | 20,20 ± 1,85 | 20,50 ± 3,25 | 19,70 ± 2,70 | 0,5688 |
| tCO_2 (_mmol/L) | 17,75 ± 2,55 | 18,45 ± 1,87 | 18,30 ± 2,10 | 0,5830 |
| EB (mEq/L) | -2,50 ± 0,70 | -2,80 ± 3,20 | -4,00 ± 0,20 | 0,1592 |
| SO_2, em %_ | 64,90 ± 17,85 | 73,65 ± 12,40 | 68,50 ± 19,20 | 0,4517 |
| Na^+^, mmol/L | 148,65 ± 3,62 | 147,20 ± 4,40 | 148,20 ± 6,70 | 0,7919 |
| K^+^, mmol/L | 3,94 ± 0,28 | 3,20 ± 0,84 | 3,95 ± 0,40 | 0,0893 |
| Ca^+2^, mmol/L | 1,29 ± 0,09 | 0,93 ± 0,81 | 1,23 ± 0,52 | 0,6632 |
| Cl^-^, mmol/L | 114,30 ± 2,55 | 119,90 ± 13,02 | 112,10 ± 5,30 | 0,4809 |

Lowercase superscript letters on the same line indicate significant differences according to the Kruskal-Wallis test and Dunn's post-test. The classification of occult blood, transitional cells, renal cells, hyaline casts, granular casts, and bacteriuria was rated as 0: absent, 1: discrete, 2: moderate, and 3: severe. Classification of transitional cells, renal cells, hyaline casts, granular casts, and bacteriuria were graded as follows: 0: absent, 1: mild, 2: moderate, and 3: severe. Pyuria and Hematuria was categorized as follows: 0: absent, 1: mild (0- cells per field); 2: moderate (6-15 red cells per field); 4: severe (>15 cells per field). pH: hydrogen potential, PCO_2_: partial pressure of carbon dioxide, PO_2_: partial pressure of oxygen, cHCO3: bicarbonate concentratio, tCO_2_: total carbon dioxide, BE: base excess, SO_2_: oxygen saturation, Na: sodium, Ca^+2^: ionized calcium, Cl^-^: chloride.

**
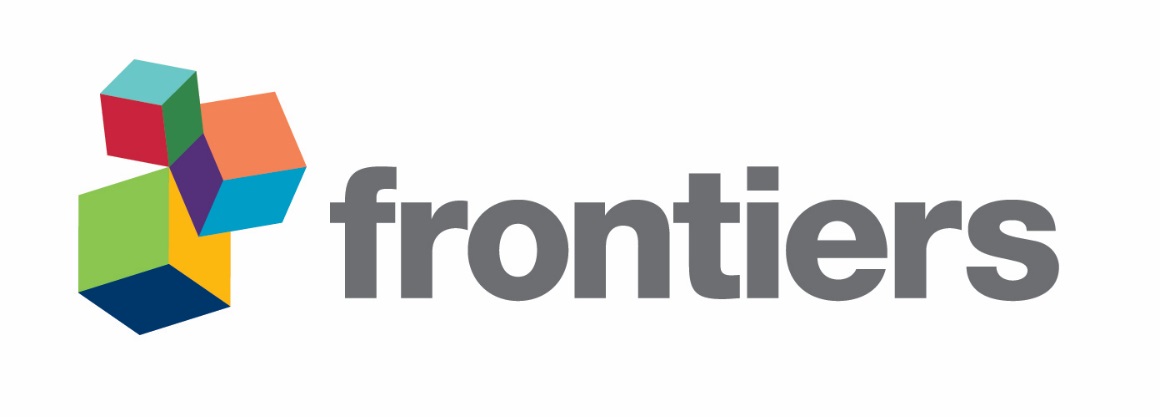
**
